# The *Babesia bovis* gene and promoter model: an update from full-length EST analysis

**DOI:** 10.1186/1471-2164-15-678

**Published:** 2014-08-13

**Authors:** Junya Yamagishi, Hiroyuki Wakaguri, Naoaki Yokoyama, Riu Yamashita, Yutaka Suzuki, Xuenan Xuan, Ikuo Igarashi

**Affiliations:** Tohoku Medical Megabank Organization, Tohoku University, 6-3-09, aza Aoba, Sendai, Miyagi 980-8579 Japan; National Research Center for Protozoan Diseases, Obihiro University of Agriculture and Veterinary Medicine, Inada-cho west 2-13, Obihiro, Hokkaido 080-8555 Japan; Department of Medical Genome Sciences, University of Tokyo, 5-1-5 Kashiwanoha, Kashiwa, Chiba 277-8562 Japan

**Keywords:** Babesia bovis, Expressed sequence tags, Full-length cDNA, Transcription start sites, Cis-elements

## Abstract

**Background:**

*Babesia bovis* is an apicomplexan parasite that causes babesiosis in infected cattle. Genomes of pathogens contain promising information that can facilitate the development of methods for controlling infections. Although the genome of *B. bovis* is publically available, annotated gene models are not highly reliable prior to experimental validation. Therefore, we validated a preproposed gene model of *B. bovis* and extended the associated annotations on the basis of experimentally obtained full-length expressed sequence tags (ESTs).

**Results:**

From *in vitro* cultured merozoites, 12,286 clones harboring full-length cDNAs were sequenced from both ends using the Sanger method, and 6,787 full-length cDNAs were assembled. These were then clustered, and a nonredundant referential data set of 2,115 full-length cDNA sequences was constructed. The comparison of the preproposed gene model with our data set identified 310 identical genes, 342 almost identical genes, 1,054 genes with potential structural inconsistencies, and 409 novel genes. The median length of 5*'* untranslated regions (UTRs) was 152 nt. Subsequently, we identified 4,086 transcription start sites (TSSs) and 2,023 transcriptionally active regions (TARs) by examining 5*'* ESTs. We identified ATGGGG and CCCCAT sites as consensus motifs in TARs that were distributed around -50 bp from TSSs. In addition, we found ACACA, TGTGT, and TATAT sites, which were distributed periodically around TSSs in cycles of approximately 150 bp. Moreover, related periodical distributions were not observed in mammalian promoter regions.

**Conclusions:**

The observations in this study indicate the utility of integrated bioinformatics and experimental data for improving genome annotations. In particular, full-length cDNAs with one-base resolution for TSSs enabled the identification of consensus motifs in promoter sequences and demonstrated clear distributions of identified motifs. These observations allowed the illustration of a model promoter composition, which supports the differences in transcriptional regulation frameworks between apicomplexan parasites and mammals.

**Electronic supplementary material:**

The online version of this article (doi:10.1186/1471-2164-15-678) contains supplementary material, which is available to authorized users.

## Background

Bovine babesiosis is a parasitic infection caused by a protozoan of the genus *Babesia*, order Piroplasmida, phylum Apicomplexa. *Babesia bovis* and *Babesia bigemina* are major species that impose a considerable economic burden on cattle industries because of their wide geographical distribution and pathogenicity [1]. The clinical symptoms of *B. bovis* are more serious than those of *B. bigemina*, including fever, extensive erythrocyte lysis leading to anemia, icterus, hemoglobinuria, and death. Although antiparasitic drugs such as imidocarb successfully control these symptoms [2], they have severe side effects and may promote the emergence of resistant strains and residual chemicals. Therefore, safer chemical agents and vaccinations are required.

In general, the genome is an excellent tool for understanding all life forms. Unique genes and pathways that are elucidated from genomes are often recognized as targets for chemical or vaccine development. Because the genome sequence of *B. bovis* is publically available [3], it may offer promising information for the development of novel approaches for controlling parasitic infections. According to a previous bioinformatics study, the *B. bovis* genome encodes 3,671 nuclear protein-coding genes. However, estimated gene models based on bioinformatics lack accuracy in nonmodel organisms. Inconsistencies in gene models have been reported between bioinformatics estimates and experimental observations of apicomplexan parasites [4, 5]. Therefore, to improve reliability, gene models require verification with experimental evidence.

The acquisition of mRNA sequences is one of the most straightforward strategies for verifying gene models. Specifically, full-length cDNA libraries facilitate the identification of transcription start sites (TSSs), exon and intron structures, 5′ and 3′ untranslated regions (UTRs), and polyadenylation sites. Moreover, massive sets of TSSs can be used to identify transcriptionally active regions (TARs), which are closely related to promoter regions [6, 7]. Therefore, the determination of full-length cDNA transcriptomes is critical for revisions of gene models, and for elucidation of transcriptional mechanisms.

In this study, we collected 5′ and 3′ expressed sequence tags (ESTs) from full-length cDNAs of *B. bovis* that were synthesized using the oligo-capping method [8]. In brief, cap structures at 5′ ends of mRNA were replaced with synthetic linker RNA sequences using the oligo-capping method. Subsequently, chimeric RNA was used to synthesize cDNA with fixed 5′ transcript sequences. This cDNA was then sequenced, and the data was entered into an updated gene model to identify novel genes. In addition, consensus sequences around TSSs and putative DNA cis-elements for transcriptional control were identified by comparison with promoter regions identified in genome-wide analyses.

## Results and discussion

### Construction and analysis of full-length cDNA

A total of 12,286 clones were randomly selected for plasmid extraction (Table 1). Subsequent one-pass sequencing from 5′ and 3′ ends using the Sanger method produced 9,573 and 10,956 sequences, respectively (DDBJ: HX874250-HX894778). After assembly of paired 5′ and 3′ ESTs using Cap3, 7,797 sequences were successfully united into one sequence, and one-pass sequences with poor quality and genes with long transcripts were excluded by miss assembly. Finally, 6,787 sequences passed the filter for coding capacity and were selected. These were annotated and redundancy was eliminated, resulting in 2,115 full-length cDNA sequences (DDBJ: AK440354–AK442468), including 1,706 cDNAs that corresponded with preproposed gene models in PiroplasmaDB, and 409 newly annotated genes (Table 1 and Additional file 1: Table S1). Among the 409 newly annotated genes, 134 showed sufficient homology to genes of other apicomplexan parasites (Additional file 1: Table S1B). In addition, features of these 134 cDNA sequences were sufficiently similar to those of the other gene sets (Additional file 2: Table S2), indicating that they may be newly identified protein coding transcripts. Among these, numbers of the genes with multiple exons and average exon numbers per gene were higher than those in other gene models (Additional file 2: Table S2), indicating that genes with multiple exons are relatively difficult to predict from genome sequences and result in miss annotation. In contrast, 273 cDNA sequences with little homology showed unique features. Specifically, the median coding sequence (CDS) length was shorter, as indicated by the smaller numbers of genes with multiple exons and longer median exon lengths than those in other gene sets (Additional file 2: Table S2). These observations suggest that certain parts of the transcripts identified in this EST analysis are noncoding RNA, or were derived from genomic DNA as artifacts. Nonetheless, promising protein coding cDNA sequences with large CDS lengths and multiple exons such as XBBk025260.contig, XBBk029358.contig, and XBBk014264.contig remained in this gene set. These *B. bovis*-specific novel genes may have *B. bovis*-specific functions in proliferation and host–parasite interactions. In general, gene finding algorithms such as GlimmerHMM [9] require training data sets for better prediction. Although training data sets for model organisms have been constructed using experimental data, available *Babesia* spp. training data sets are limited, potentially reflecting the observed discrepancies between experimentally observed cDNAs and preproposed gene models. Because a degree of consistency was observed between the 1,706 full-length cDNA sequences and preproposed annotations, we performed genome and amino acid alignments of these sequences (Table 1). In these analyses, 310 sequences were identical to preproposed genes, whereas 342 were almost identical but with amino acid substitutions that probably originated from sequencing errors or polymorphisms among strains. The remaining 1,054 sequences had partial homology to existing annotations, although they had structural inconsistencies that may reflect the alternative usage of start codons and/or splicing.

**Table 1:** **Summary of ESTs and contigs**

	Number	Accession number
Total number of isolated clones	12286	
5*'* one-pass sequence	9573	DDBJ: HX874250-HX894778
3*'* one-pass sequence	10956	
Contig sequence	6787	
Non-redundant contig sequence^1)^	2115	DDBJ: AK440354-AK442468
Identical^2)^	310	
Amino acid variant^3)^	342	
Structural variant^4)^	1054	
Assigned in this study^5)^	409	

1) Nonredundant contig sequences were selected from the contig sequence. Identical, amino acid, structural, and assigned variants were subsets of nonredundant contig sequences. 2) Contig sequences with identical coding sequences to the preproposed gene model (ppgm); 3) Contig sequences with almost identical coding sequence but amino acid variant(s) derived from single nucleotide variant(s); 4) Contig sequences with structural differences to that of the ppgm assigned in this study; 5) Contig sequences not described in the ppgm.

The 5′ UTRs that lie between TSSs and first in-frame initiation codons are known to play crucial roles in post-transcriptional regulation by modulating translational efficiency and mRNA stability through the actions of IRES and riboswitches [10, 11]. This mechanism is observed in a wide variety of organisms, including humans, plants, and yeast [12–14], suggesting that apicomplexan parasites have similar functions. However, these functions have been poorly investigated. Therefore, to elucidate the functions of the 5′ UTRs of *B. bovis*, we constructed a genome-wide 5′ UTR sequence data set using full-length cDNA sequences and demonstrated that the median length of the 5′ UTRs of *B. bovis* is 152 nts. The average 5′ UTRs are 210.2 nts in humans, 186.3 nts in rodents, 221.9 nts in invertebrates, 103.0 nts in viridiplantae, and 134.0 nts in fungi [15] and the mode length is approximately 130 nts in *Toxoplasma gondii* [16]. These lengths agree with our observations in *B. bovis*. Similarly, the median length of the 3′ UTRs of *B. bovis* is 116 nts (Additional file 2: Table S2).

Gene expression frequencies are also indicated in EST data. Therefore, we examined the 9,573 5′ ESTs data set and selected 9,546 sequences following successful mapping onto the *B. bovis* genome. To estimate expression frequencies, these were then mapped onto preproposed CDSs with novel sequences identified in this study (Additional file 3: Table S3 and Additional file 4: Figure S1). The resulting ranking was not identical to that in a previous study of ESTs [17], although it showed similar tendencies. These discrepancies may reflect differences in culture conditions and parasite strains or sampling errors associated with small data sets. Logarithmic plots of expression levels and ranks of each gene resembled the power law (Additional file 4: Figure S1) and indicated similar transcriptome distributions to those observed in previous studies [18, 19].

### *B. bovis*promoter components and typical structure

Transcription is controlled by the coordinated binding of promoter sequences by transactivators. In humans and model organisms, promoter structures have been intensively examined in a genome-wide manner [20–22] and have been shown to play pivotal roles in gene and phenotype expression. However, the promoter structure of *Babesia* spp. remains unknown. Therefore, we characterized the promoter structure of *B. bovis* using high resolution TSS information derived from a full-length cDNA data set.

Genome-wide TSS distributions were examined by mapping 5′ ends of 5′ ESTs. In briefly, 9,412 reliable 5′ end sequences of 36 nts were selected from 9,573 5′ ESTs. Of these, 7,111 were successfully mapped onto the *B. bovis* genome sequence, 4,086 locations were assigned as TSSs after considering redundantly mapped sequences, and 2,023 TARs were identified.

**Figure 1:**
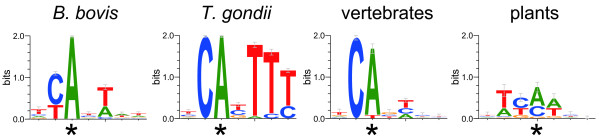
**Consensus sequences on TSSs.** Asterisks indicate TSSs.

**Figure 2:**
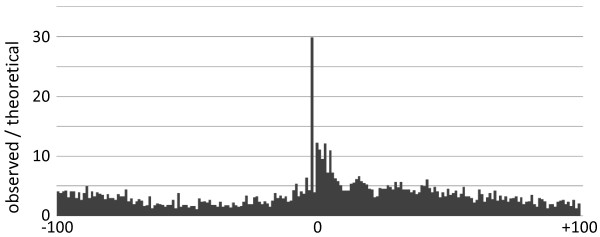
**Distribution of the TYAYWWW motif around TSSs.** The horizontal axis represents sequence areas from -100 to +100 around TSSs with 1-nt resolution. Position 0 represents TSS. A peak was observed at the -2 position. The vertical axis represents the ratio of the motif frequency and the theoretical frequency (see Methods).

We selected motifs in the -10 to +10 regions of TSSs from the TAR data set and examined these using MEME [23]. The estimated consensus sequence TYAYWWW was found in 801 of the 2,023 TARs, with p values of <0.05 (Table 2 and Figure 1). We also examined the positional distribution of this motif around TSSs. Examinations of sequences around TSSs (-100 to +100 region) showed that the motif was distributed only on TSSs (Figure 2). Moreover, adenine residues at TSSs and cytosine residues at the -1 position were clearly conserved and +3 to +5 positions tended to be thymidine, as shown in *T. gondii* [7]. This CA motif was also conserved in initiator consensus sequences from vertebrates [24] and dicotyledonous plants [25], despite differences in the methods for identifying consensus and diversity of subject species. Data sets for *B. bovis* and *T. gondii* were collected from single organisms, whereas the data sets from vertebrates and plants were collected from multiple organisms. According to molecular recognition analyses, the initiators TAF1 and TAF2 play pivotal roles [26–28]. In *Plasmodium falciparum*, PFL1645w and MAL7P1.134 are promising functional homologues of TAF1 and TAF2, respectively, as predicted using bioinformatics methods [29]. Moreover, their corresponding genes BBOV_IV004260 and BBOV_II003570 were annotated in the *B. bovis* genome, implying that initiator recognition and TSSs have evolved with closely related molecular mechanisms across taxonomic kingdoms.

**Table 2:** **FWM for** ***B. bovis*** **initiator-like motifs from 801 TSSs**

Position from TSSs	-2	-1	0 ^*^	+1	+2	+3	+4
A	142	0	801	109	270	308	234
T	411	245	0	326	480	255	325
G	104	0	0	133	0	127	137
C	144	556	0	233	51	111	105
consensus	T	Y	A	Y	W	W	W

*position of TSS.

In subsequent analyses, we identified a cis-element that is involved in transcriptional control. To generate a putative promoter set, -1000 to +1000 regions from typical TSSs of the 2,023 TARs were selected and examined using CisFinder [30], and -100–0 regions were examined using MEME [23]. These analyses showed frequent distribution of ATGGGG and ACACA within promoter regions.

**Figure 3:**
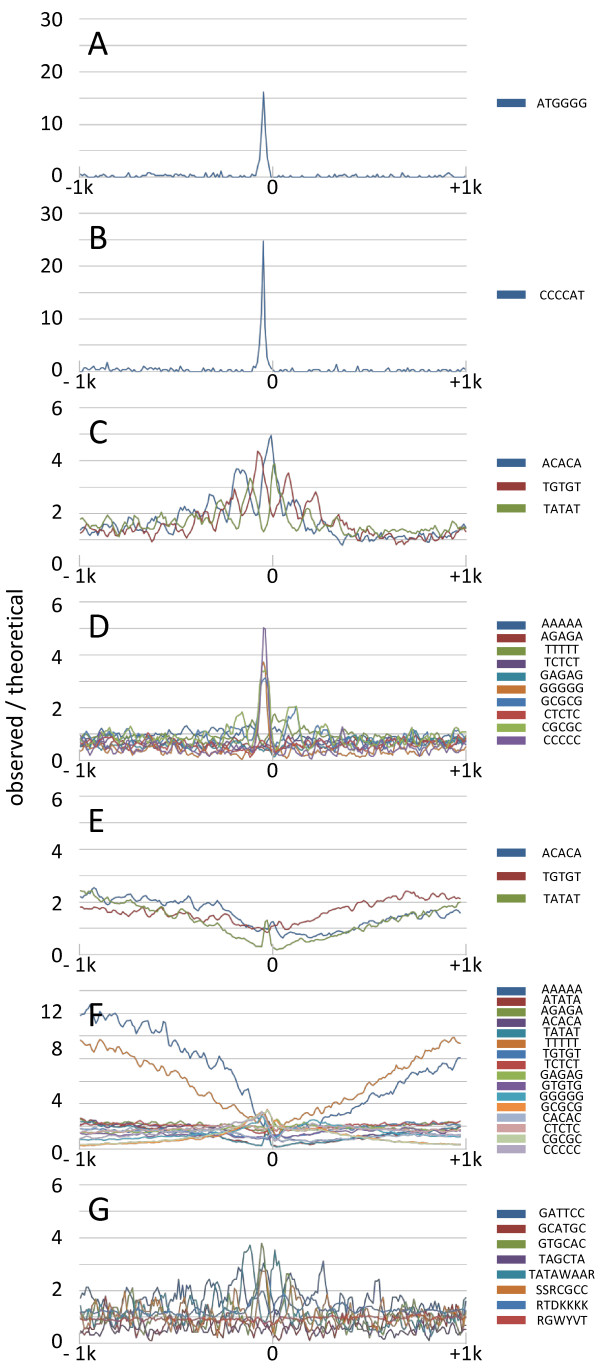
**Distributions of motifs around TSSs.** Horizontal axes represent sequence areas from -1000 to +1000 from TSSs. Vertical axes represent the ratio of motif frequencies and theoretical frequencies (see Methods). **(A)** Distributions of the ATGGGG motif; **(B)** Distribution of the CCCCAT motif; **(C)** Distributions of the ACACA (blue), TGTGT (red), and TATAT (green) motifs; **(D)** Distributions of the other 5-mer motifs; **(E)** Distributions of the ACACA (blue), TGTGT (red), and TATAT (green) motifs in humans; **(F)** Distributions of all 5-mer repeat combinations in humans; **(G)** Distributions of the known apicomplexan motifs GATTCC, GCATGC, GTGCAC, and TAGCTA and the core mammalian promoter motifs TATA, TATAWAAR; BRE^u^, SSRCGCC; BRE^d^, RTDKKKK; and the DPE, RGWYVT. In **A**, **B**, and **G**, the scanning window was 10 bp. In **C–F** the scanning window was 30 bp.

To validate the ATGGGG motif, we examined positional distributions of these candidates around TSSs and found a clear peak at 50 nts upstream (Figure 3A). Further investigations of the reciprocal sequence CCCCAT showed equivalent distribution to that of ATGGGG (Figure 3B), implying that the motif may be functional regardless of its direction. The CCCCAT motif has been identified in *Theileria parva* and *Theileria annulata* using encyclopedic promoter analyses. Although the reciprocal motif ATGGGG was not examined in these species, its peak was found at -20 nts from TSSs, differing slightly from our observations [31]. In further investigations, we examined functional enrichments of genes carrying these promoter motifs, and identified genes corresponding to the 2,023 TARs by calculating relative distances. Subsequently, 1,315 TARs were found with candidate initiation codons. Among these, 222 TARs had the ATGGGG or CCCCAT motifs in the -80 to -20 region from TSSs. Subsequent enrichment analyses using gene ontology terms from GOstat [32] indicated significant enrichment in “structural constituent of ribosome” (GO:0003735) and “translation” (GO:0009058) categories, with E-values of 3.43e^-08^ and 2.06e^-06^, respectively. Enrichments of protein synthesis have also been reported for the CCCCAT motif in *T. parva* and *T. annulata* [31], suggesting that the motif may be conserved in piroplasms as a transcriptional regulator of genes involved in protein synthesis.

To validate the ACACA motif, we examined the positional distribution of these candidates and found periodical distribution around TSSs (Figure 3C). The reciprocal sequence TGTGT was also periodically distributed, but its phase was shifted (Figure 3C). Based on these observations, we examined all 5-mer repeat motifs comprising two nucleotides and found periodical distribution of TATAT as an additional motif (Figure 3C), with similar cycles but differing phases (Figure 3C). The related motifs CACAC, GTGTG, and ATATA also showed similar distributions (data not shown). The only other combinations that showed distinguishing distributions were GGGGG, CCCCC, GCGCG, and CGCGC, with peaks around -50 nts (Figure 3D). GGGGG and CCCCC motifs are closely related to ATGGGG and CCCCAT motifs, respectively. However, GCGCG and CGCGC motifs may be functional and gene ontology enrichment analyses showed frequent but insignificant presence of these in upstream promoter regions of ribonucleoprotein complex biogenesis (GO:0022613) genes (p = 0.078). To confirm the specificity of these motifs for apicomplexan parasites, we examined periodical distributions in the TSS database DBTSS, which contains precise positions of TSSs in the genomes of various organisms [33]. Promoter regions from -1000 to +1000 of human and mouse TSSs were obtained and the distribution of ACACA, TGTGT, and TATAT motifs were examined as in *B. bovis*. However, no periodical distributions were found in human (Figure 3E) or mouse (data not shown) databases, and no related periodical distributions of other combinations were observed as in *B. bovis* (Figure 3F). In contrast, the ACACA motif was reportedly observed in *T. parva* and *P. falciparum* [31, 34], although periodical distributions have not been reported. Rather than reflecting the differences in species, these discrepancies may have been caused by differences in the precision of TSS identification. Nonetheless, these observations imply that the motif is common among some apicomplexan parasites, and the present periodical patterns had interval lengths of 140–150 nts. Minimum units of nucleosome repeat lengths comprise 147-bp DNA sequences around core histone octamers and 20-bp DNA linkers and are much longer than our observations. However, previous studies demonstrate that the minimum observed nucleosome repeat length is much closer to our observation of approximately 155 bp in *Schizosaccharomyces pombe* and *Aspergillus nidulans* [35–37], and *P. falciparum* [38, 39]. On the other hand, these discrepancies may reflect the involvement of unconventional nucleosome structures. The conventional histone octomer comprises two H2A, H2B dimers and H3, H4 tetramers. In contrast, unconventional histones comprising variants such as H2B.Z, H2A.Z, and CENP-A have specific functions that are distinguishable from the conventional one. Crystal structure analysis of human centromeric nucleosomes containing CENP-A suggests that only 121-bp DNA fragments tightly bind to nucleosomes, unlike conventional H3 nucleosomes [40]. In *P. falciparum*, it was demonstrated that the nucleosome with H2A.Z specifically localizes to intergenic regions [41, 42]. Moreover, no homologue to the linker histone H1 has been identified in apicomplexan parasites [43]. FAIRE-seq and MAINE-seq analyses in *P. falciparum* demonstrated that nucleosome binding to TSSs is associated with gene expression [44] and there are preferred DNA motifs for nucleosome assembly [45, 46]. These collateral data warrant the assumption that the observed periodical patterns in this study are involved in chromatin structure and regulate gene expression via chromatin remodeling processes.

In further analyses, we applied this scanning method to known apicomplexan and mammalian core promoter motifs. A previous study showed the distribution of GATTCC in *T. parva* and *T. annulata* at regions that are -20 nts from TSS [31]. Moreover, GCATGC was identified as a PF14_0633-binding target in *P. falciparum* [47], an AP2-Sp-binding target in *Plasmodium berghei* [48], and a Toxoplasma Ribosomal Protein (TRP)-2-binding target in *T. gondii* [49]. GTGCAC is known as a subtelomeric variant gene promoter element (SPE)-2 [49] and a binding target of PFF0200c_DLD and PfSIP2 [47, 50, 51]. TAGCTA is also reportedly a binding target of Pb.AP2-O [52]. Therefore, although the GTGCAC motif was moderately concentrated in the -50-nt area, the other motifs did not show distinguishing distributions in comparison with the ATGGGG/CCCCAT motif (Figure 3G). In particular, GATTCC was not specifically distributed around TSSs, as observed in *T. parva* and *T. annulata,* indicating that the motif is specific to *Theileria* spp. and may be involved in specific biological phenotypes, such as infectivity in lymphocytes. According to mammalian motifs, we examined TATA boxes, upstream TFIIB-recognition elements (BRE^u^), downstream BRE (BRE^d^), and downstream promoter elements (DPE), containing the consensus sequences TATAWAAR, SSRCGCC, RTDKKKK, and RGWYVT, respectively [20]. In these analyses, TATA boxes showed periodical-like patterns (Figure 3G). In contrast, TATA boxes are known to be distributed around -30 nts from TSSs (Figure 3E), and TATA box consensus sequences are closely related to TATAT and ATATA motifs. These are also periodically distributed, suggesting that the observed pattern for TATA boxes was residual and no functional motifs correspond with the TATA box in *B. bovis*. This observation also indicates that other mammalian motifs are nonfunctional (Figure 3G).

**Figure 4:**
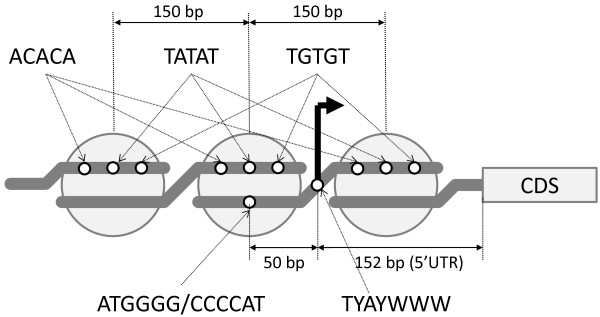
**Schematic representation of the composition and speculative nucleosome structure of a model** ***B. bovis*** **promoter.** Angled arrows, thick lines, big circles, and boxes represent TSSs, DNA, histones, and coding sequences, respectively. Motifs in promoter regions are represented by small circles and the corresponding sequences are indicated by dashed arrows. The TYAYWWW motif over TSS is shown in Figures 1 and 2, and Table 2. The median length from TSS to the 5*'* end of CDS (5*'* UTR) was 152 bp (Additional file 2: Table S2). The ATGGGG motif and its reciprocal CCCCAT are distributed around -50 nt from TSS (Figure 3A and B). The ACACA, TGTGT, and TATAT motifs appear every 150 bp (Figure 3C). Positional relationships among the motifs and histones are arbitrarily described in this illustration.

Collectively, we speculate model promoter structures and transcriptional mechanisms in *B. bovis* that explain our observations (Figure 4). Primarily, we identified the TSS initiator-like motif TYAYWWW. In other taxonomic kingdoms, this initiator works as a binding site for the general transcription factors TAF1 and TAF2 [20], and previous *in silico* analyses demonstrate that apicomplexan parasites express homologs of TAF1 and TAF2 [29]. Therefore, *B. bovis* may also use this molecular mechanism at the final step of transcriptional initiation, as described previously in *P. falciparum* [53]. Similar to *T. gondii* and majority of other organisms, the average length of 5′ UTRs was 150 nts, suggesting similar involvement in the regulation of gene expression, similar to that in other organisms. Periodical distributions of ACACA, TGTGT, and TATAT were observed around TSSs. However, this profile was not observed in human and mouse (Figure 3), and previous studies indicate that transcriptional mechanisms differ between apicomplexan parasites and other eukaryotes to a certain degree [43, 53, 54]. In particular, we assumed that the periodical distributions are involved in tight assembly of nucleosome structures and control transcription, although discrepancies of nucleosome repeat lengths remains to be clarified by additional experimental evidences. On the other hand, we observed clear peak distributions of ATGGGG and CCCCAT at -50-bp regions from TSSs. Although it remains unclear how this motif functions regardless of orientation, chromatin remodeling factors may be recruited to loosen nucleosome structures. Therefore, the scheme shown in Figure 4 proposes transcriptional arrest by histones and subsequent activation by putative chromatin remodeling factors that interact with ATGGGG or CCCCAT elements.

Previous investigations of *Plasmodium* and *Toxoplasma* demonstrate promoter structures [43, 53–55], putative DNA cis-elements [34, 44, 50, 54, 56–58], and the involvement of chromatin structures in transcription [44, 53, 55, 59]. The present analyses of *Babesia* parasites were almost consistent with these studies and warrant the expansion of the concepts related to *Babesia* species. Nonetheless, the use of fine TSS mapping is a critical distinction between the present and previous studies and allowed more specific and sensitive assessment of the distribution of examined motifs, particularly for ACACA, TGTGT, and TATAT motifs that lack definition in previous studies [34, 50]. Therefore, the present analyses indicate that the distance from TSSs may be a critical factor for functionality of DNA cis-elements in apicomplexsan parasites.

## Conclusions

The full-length cDNAs dataset enable us to revise previous gene model derived from the genome. In parallel, location-specific consensus motifs in promoter sequences were discovered by virtue of TSSs identification with one-base resolution of the method. These observations 1) indicate the utility of integrated bioinformatics and experimental data for improving genome annotations and 2) allowed the illustration of a model promoter composition, which supports the differences in transcriptional regulation frameworks between apicomplexan parasites and mammals.

## Methods

### Preparation of parasite RNA, and synthesis and sequencing of cDNA

The Texas strain of *B. bovis* was maintained in bovine erythrocytes cultured in GIT medium (WAKO, Osaka, Japan) using a microaerophilic stationary-phase culture system [60]. Total RNA was extracted from *B. bovis*-infected erythrocytes using TRIzol (Invitrogen), and cDNA was synthesized using a previously described oligo-capping method [61]. In briefly, 200 μg of purified total RNA was dephosphorylated using bacterial alkaline phosphatase and was ligated using the oligo-RNA 5′-AGCAUCGAGUCGGCCUUGUUGGCCUACUGG-3′ and T4 RNA ligase. Subsequently, cDNA was synthesized using the oligo-dT fusion primer 5′-GCGGCTGAAGACGGCCTATGTGGCCTTTTTTTTTTTTTTTTT-3′ with SuperScript® II (Invitrogen). The cDNA library was amplified using PCR with the primers 5′-AGCATCGAGTCGGCCTTGTTG-3′ and 5′-GCGGCTGAAGACGGCCTATGT-3′. Amplified fragments were then digested using SfiI and were ligated into a DraIII-digested pME18SFL3 plasmid vector in an orientation-defined manner. ESTs of 5′ and 3′ ends were obtained using the Sanger method with ABI 3730 sequencers following standard protocols for sequencing analysis.

### Assembly, clustering, and annotation of ESTs

To obtain full-length cDNA sequences, 5′ and 3′ ESTs were assembled using a CAP3 [62] with default parameters. The overlapping nucleic acid length cutoff was 40 and the overlapping identity cutoff was 90% for 5′ and 3′ ESTs, respectively. Putative CDSs of full-length cDNA were examined and intact CDSs of >50 amino acids were selected. Amino acid homology with preproposed genes (BbovisT2BoAnnotatedProteins_PiroplasmaDB-1.1.fasta) was examined using BLAST and homology was considered significant when E-values were 10^-10^. Full-length cDNA sequences were also mapped onto the genome of *B. bovis* (BbovisT2BoGenomic_PiroplasmaDB-1.1.fasta) using BLAT [63] with default parameters. The categories of full-length cDNA sequences (tier 1; identical, tier 2; amino acid variant, tier 3; structural variant, and tier 4; novel) were assigned according to BLAST and BLAT results using an in-house script. Copy DNAs that were mapped to positions of identical nucleic acid sequences of preproposed genes were assigned as “identical”, and those mapped to identical positions but with amino acid substitutions were assigned as “amino acid variants”. Copy DNAs that were mapped to similar but nonidentical positions to homologous preproposed genes were assigned as “structural variants”, and those with poor homology were assigned as “novel”. Full-length cDNAs were clustered using CAP3 with default parameters, and the subset of cDNAs in the same cluster were integrated into the highest tier. Among full-length cDNAs with differing sequences and the same tier status, cDNAs with fewer mutations and longer amino acids or nucleic acids were selected, and novel cDNAs were annotated using Blast2Go [64]. To estimate gene expression, 5′ ESTs were examined. Initially, these were filtered by BLAST using the *B. bovis* genome database (BbovisT2BoGenomic). Subsequently, the filtered sequences were mapped onto both CDS (BbovisT2BoAnnotatedCDS) and novel sequences using BLAST, and the frequencies of mapped ESTs were determined.

### Identification of promoter regions and prevalent motifs

To identify TSSs for each 5′ EST, 36 nts were clipped from 5′ ends and removed if they contained ambiguous nucleotides such as N. Selected sequence sets were then mapped onto the genome sequence of the *B. bovis* T2Bo strain [3] using Bowtie [65] with the acceptance of two mismatches. To assign TARs, mapped positions corresponding with TSSs were clustered if two TSSs were positioned within 20 nts. Gross mapped counts for each position and TAR were tallied, and the most frequently mapped TSS in each TAR was assigned as a representative TSS, as defined in previous reports [33, 66]. To identify motifs on TSSs, -10 to +10 regions from representative TSSs were selected and examined using MEME [23]. A frequency weight matrix (FWM) was calculated on the basis of sequence motifs with p values of <0.05, and a sequence logo was generated on the basis of FWM using WebLogo [67]. As a putative promoter region, sequences comprising -1000 to +1000 regions from representative TSSs of each TAR were selected. Human and mouse promoter regions were obtained from DBTSS [33]. Candidate DNA motifs were then estimated using CisFinder [30] with default parameters and -1000 to +1000 regions from representative TSSs. These were also estimated using MEME with n sites of 2023, maximum DNA sizes of 250000, and maxw of 8 as parameters in -100–0 regions of representative TSSs. The distributions of identified motifs around peak TSSs were examined by scanning the motif over the promoter using an in-house script. The positions of each motif were scanned in exact match condition and summed for every 10 bases. Observed frequencies were divided by the theoretical frequency based on nucleotide biases that were estimated from the nucleotide composition of the genome. The distributions of 5-mer motif candidates were smoothed by averaging the surrounding 30 bases. For functional enrichment analyses, we selected TARs with motifs from the initial TAR set of 2,023-nt sequences. Genes and TARs were linked if TARs were present in the -500 to +200 region from the 5′ end of the CDS, as defined in the BbovisT2BoAnnotatedCDS. Finally, gene ontology terms for *B. bovis* genes were annotated using Blast2Go [64].

### Availability of supporting data

Supporting sequence data are available in the DDBJ (http://www.ddbj.nig.ac.jp/index-e.html) under accession numbers HX874250–HX894778 and AK440354–AK442468.

## Authors’ information

This paper is dedicated to the memory of Dr. Junichi Watanabe.

## Electronic supplementary material

Additional file 1: Table S1.: Analyzed sequences. (A) Nonredundant full-length cDNA sequences with corresponding *B. bovis* genes; 5′ UTR lengths, isolated sequences, and coding sequences (amino acid and nucleotide sequences) that corresponding with genes; (B) Nonredundant full-length cDNA sequences that were newly annotated in this study; Results from a BLAST search and InterProScan were added. (XLSX 2 MB)

Additional file 2: Table S2.: Model comparison of pre-proposed genes with those identified in this study. (XLSX 11 KB)

Additional file 3: Table S3.: Frequency of gene expression in *B. bovis*. (XLSX 84 KB)

Additional file 4: Figure S1.: Statistical profile of ESTs in *B. bovis.* Counts of ESTs that encode the same genes were converted to a logarithm and were plotted on the horizontal axis. The ranks of EST counts were converted to a logarithm and were plotted on the vertical axis. (PDF 309 KB)
