# Supplementary figures and images for: The Babesia bovis gene and promoter model: an update from full-length EST analysis
Source: BMC Genomics. 2014 Aug 13;15(1):678. doi: 10.1186/1471-2164-15-678 (PMC4148916; doi:10.1186/1471-2164-15-678)

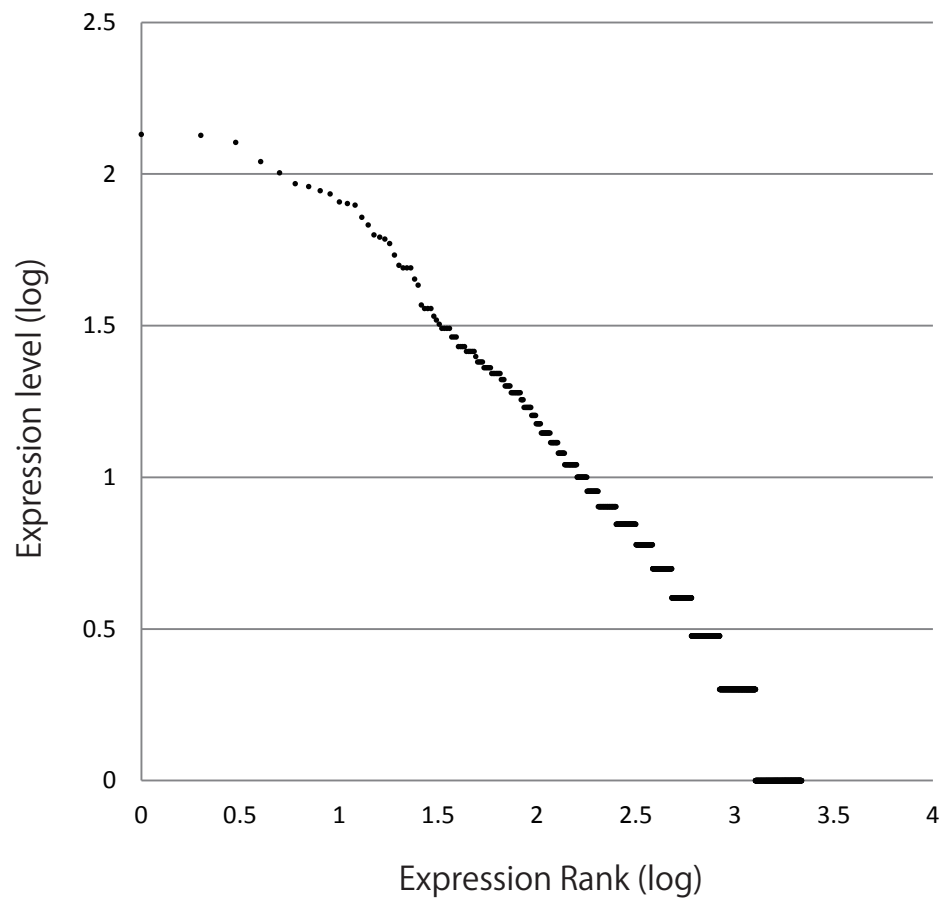

Fig. SX

Supplement: Supplementary file 4 — Additional file 4: Figure S1.: Statistical profile of ESTs in B. bovis. Counts of ESTs that encode the same genes were converted to a logarithm and were plotted on the horizontal axis. The ranks of EST counts were converted to a logarithm and were plotted on the vertical axis. (PDF 309 KB) [file 12864_2013_6376_MOESM4_ESM.pdf]
